# Improvement of K-Means Algorithm and Its Application in Air Passenger Grouping

**DOI:** 10.1155/2022/3958423

**Published:** 2022-09-12

**Authors:** Donghua Yu, Shuhua Dong, Shuang Yao

**Affiliations:** ^1^Department of Computer Science and Engineering, Shaoxing University, Shaoxing 312000, China; ^2^College of Economics and Management, China Jiliang University, Hangzhou, China

## Abstract

The k-means is one of the most popular clustering analysis algorithm and widely used in various fields. Nevertheless, it continues to have some shortcomings, for example, extremely sensitive to the initial center points selection and the special points such as noise or outliers. Therefore, this paper proposed initial center points' selection optimization and phased assignment optimization to improve the k-means algorithm. The experimental results on 15 real-world and 10 synthetic datasets show that the improved k-means outperforms its main competitor k-means ++ and under the same setting conditions, namely, using the default parameters,its clustering performance is better than Affinity Propagation, Mean Shift, and DBSCAN. The proposed algorithm was applied to analyze the airline seat selection data to air passengers grouping. The clustering results, as well as absolute deviation rate analysis, realized customer grouping and found out suitable audience group for the recommendation of seat selection services.

## 1. Introduction

Clustering is to divide the dataset into nonoverlapping subsets, such that the objects in the cluster are as similar as possible, and the objects between the clusters are as dissimilar as possible [1]. There are numerous kinds of clustering algorithms, such as AP [2], DPC [3–6], which show excellent clustering performance. However, as one of the most classic clustering algorithm, the k-means aimed to partition the given dataset into *K* subsets so as to minimize the within-cluster sum of squared distances continues to be one of the most popular clustering algorithms [7]. Its efficiency and simplicity of implementation make it successfully applied in various fields, such as image [8, 9], education [10], bioinformatics [11], medical [12], partial multiview data [13], agricultural data [14], fuzzy decision-making [15].

Optimizing the initial center points may be one of the most effective methods to improve the performance of k-means algorithm. The study of Fränti and Sieranoja [16] reported that (a) the k-means clustering algorithm can be significantly improved by using a better initialization technique and by repeating (re-starting) the algorithm; (b) when the data have overlapping clusters, k-means can improve the results of the initialization technique; (c) when the data have well separated clusters, the performance of k-means depends completely on the goodness of the initialization; (d) initialization using simple furthest point heuristic (Maxmin) reduces the clustering error of k-means from 15% to 6%, on average. With the popularity of deep learning in various fields, optimizing data representation is also a means to improve clustering performance, especially in the face of high-dimensional data. The robust deep k-means (RDKM) algorithm [17] exploit the hierarchical information of multiple-level attributes with using the deep structure to hierarchically perform k-means.

**Figure 1:**
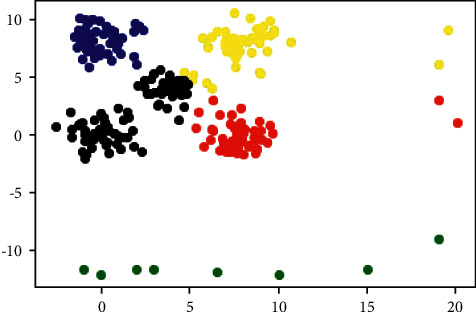
The k − means++ clustering result on synthesized dataset.

The k − means++ [18] provided a simple and effective initial center points optimization method called *D*^2^ − sampling. It adds new center point one by one and assigns different selection probabilities to each potential center point. Since then, especially after being embedded in scikit-learn as the default k-means algorithm, it has almost become the first choice based on partitioning clustering algorithms. However, due to k-means ++ randomly selects the first center point uniformly and randomly adds subsequent center points according to the probability, some special data distribution can also lead to k-means ++ poor results, even unreasonable clustering results. For example, a dataset with five clusters is synthesized and some noise points half-circle surrounding them are added. The clustering result of k-means ++ was shown in Figure 1, where each color represents a cluster. The desired clustering result should be that the points in the upper left corner are divided into five clusters, but the actual result is that the points in the lower (green points) are clustered into a single cluster to be a wrong result. In this paper, some methods were proposed to solve this problem.

Cluster analysis is one of the basic methods of data knowledge discovery. With the development of airline business, ancillary services that satisfy passengers' personal requirement are becoming more and more important for airlines [19, 20]. However, owing to the impact of COVID-19, the airline market faced a dramatic regression (2019–2021), compelling airlines to seek revenue other than from flight tickets [21, 22]. Therefore, establishing ancillary services is significantly important for airlines due to the ability to increase the airline's revenue. In this paper, the improved k-means algorithm is applied into cluster analysis an airline seat selection dataset, which aims to group airline passengers to serve the establishment of auxiliary services.

Based on the above analysis and application requirements, this paper proposed an improved k-means algorithm, called as k-means2o, based on initial center points selection optimization and phased assignment optimization, and realized the clustering analysis on airline seat selection dataset. The main contributions are summarized as follows:

1. Two optimization methods are proposed for the k-means algorithm: initial center points selection and phased assignment. In the initial center points selection optimization, this method inherits the center point incremental strategy of k-means ++ [18], K-MC ^2^ [23] and AFK-MC ^2^ [24], but redefines the first center point selection strategy and the subsequent center point incremental strategy. In the phased assignment optimization, the Tukey's rule is adopted to divide dataset into core and noncore sets to realize two-stage assignment, then two assignment strategies are proposed corresponding to the core and noncore sets, respectively.
2. Four popular algorithms, k-means ++ [18], affinity propagation [2], mean shift [25], and DBSCAN [26], are used to verify the effectiveness and the performance improvement of k-means2o based on 15 real-world and 10 synthetic datasets. Further, the impact of core and noncore sets on the clustering result is analyzed.
3. The improved k-means algorithm is applied to an airline seat selection dataset, and the passenger groups who are more willing to pay for seat selection are found out. The absolute deviation rate adr is defined to analyze the significance of passenger grouping. This provides valuable information for auxiliary services.

## 2. Related Works

There are many possible ways to optimize the initial center points. The k-means ++ [18] provided *D*^2^ − sampling method which assigns different selection probabilities to each potential center point. Bachem et al. [23] replaced the *D*^2^ − sampling in k-means ++ with MCMC-sampling and obtained a nearly linear improved k-means algorithm K-MC^2^. However, this algorithm defines two data-dependent hypothesis *α*(*X*),  *β*(*X*), which will have an important impact on the clustering result and the algorithm complexity. Subsequently, Bachem et al. [24] solved the hypothesis defect of the K-MC^2^ algorithm. They extended a regular term based on *D*^2^ − sampling of k-means ++. This new algorithm is called AFK-MC^2^. Whether it is K-MC^2^ or AFK-MC^2^, they all follow the first center point selection strategy of the k − means++ algorithm, namely that it first samples an initial center uniformly at random. At the same time, they all have similar center point selection methods, that is, a point farther from the currently selected center points has a greater probability of being chosen as the next center point. For more information on the optimization method of the initial center point, please consult the literature [27].

Phased assignment, generally speaking, is to divide the data into different stages to complete the cluster label assignment, or assign the cluster labels to only part of the data, and the remaining part will be removed as outliers, noise, etc. Zhou et al. [28] proposed a three-stage k-means algorithm to cluster data and detect outliers. In the first stage, the fuzzy c-means algorithm is applied to cluster the data. In the second stage, local outliers are identified, and the cluster centers are recalculated. In the third stage, certain clusters are merged, and global outliers are identified. Im et al. [29] proposed the NK-means algorithm which emphasizes the removal of noise/outliers and is a two-stage k-means algorithm. In the first stage, a greedy algorithm is utilized to remove abnormal points. In the second stage, the center points are optimized in the constructed core set, and cluster label is assigned to each point. In term of preprocessing techniques, k-means ++ is utilized as an additional filtering step to remove out *z* of data points as outliers before applying the conventional k-means. The clustering process is only performed on the remaining data which are outlier-free. The outliers data are completely removed and not classified to any known cluster as collected initially. The KMOR algorithm is proposed by Gan and Ng [30] assigns outliers to an additional cluster. This algorithm redefines the clustering objective function and takes into account the SSE between outliers and center points. However, it introduces two new parameters to adjust outlier number. The k-means-sharp is proposed by Olukanmi et al. [31] to eliminate the outliers' influences from the clusters' centroid. The detected outliers are completely excluded from the mean measurement only, but they are involved later in the clustering process. However, the data point with all attributes is eliminated completely from centroid measurement. In this case, the algorithm cannot recognize an outlier's presence in every attribute independently. This is because the single value of the distance metric represents the entire vector instead the single attribute be removed. Therefore, an empty cluster may occur in case of the presence of at least one outlier in each data point [32]. The phased assignment is not only used to optimize the k-means algorithm. For example, Yu et al. [33] also adopted a two-stage assignment strategy based on boundary conditions to optimize the DPC clustering algorithm. For a dataset to be clustered, in many cases, users do not care whether it contains outliers, because the outliers themselves are difficult to define, but they definitely want to assign them cluster labels. Wang et al. [34] proposed an improved integrated clustering learning strategy based on three-stage affinity propagation algorithm with density peak optimization theory (DPKT-AP). In the first stage, the clustering center point was selected by density peak clustering. In the second stage, the k-means algorithm was used to cluster the data samples. In the third stage, DPKT-AP used the AP algorithm to merge and cluster the spherical subgroups.

## 3. Proposed K-Means Algorithm

Suppose a given dataset *X*={*x*_1_, *x*_2_,…, *x*_*n*_}, *x*_*i*_ ∈ *ℝ*^*m*^, and divide it into *K* mutually disjoint sets *C*={*C*_1_,…, *C*_*K*_}, so that ∪_*i*_^*K*^*C*_*i*_=*X* and *C*_*i*_∩*C*_*j*_=Φ, ∀*i*, *j*, *i* ≠ *j*.

### 3.1. Initial Center Points Optimization

Like the k-means ++ algorithm, the k-means2o adopts a strategy of increasing center points one by one until the desired *K* points are reached. However, the difference is that the new algorithm redefines the selection of the first center point and subsequent center points. For this purpose, first, define the distance function d(*x*, *S*) between the point *x* and the set *S*:

(1) $$\begin{array}{c} \text{d}\left(x,S\right)=\min_{x_{j}\in S}\text{d}\left(x,x_{j}\right), \end{array}$$

where d(*x*, *x*_*j*_) represents the distance between two points *x*, *x*_*j*_. In this paper, Euclidean distance is selected.

Let *c*_*i*_, *i*=1,…, *K* represent the center point of cluster *c*_*i*_, *i*=1,…, *K*, then the first center point *c*_1_ is selected as follows:

(2) $$\begin{array}{c} c_{1}=\frac{1}{\left|S_{\text{core}}\right|}\sum_{x_{i}\in S_{\text{core}}}x_{i}, \end{array}$$

where |*S*_core_| represents the number of elements in the core set *S*_core_. Then, the (2) shows that *c*_1_ is the mean value of the core set *S*_core_.

**Figure 2:**
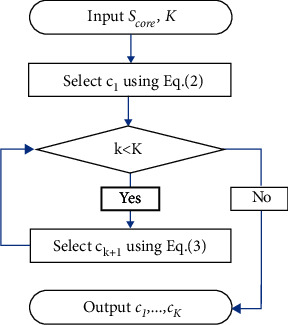
The flow chart of initial center points optimization.

Let *C*^*k*^={*c*_1_,…, *c*_*k*_} represents a set containing *k* center points, then the selection method of *k*+1 th center point *c*_*k*+1_ is as follows:

(3) $$\begin{array}{c} c_{k+1}=\text{arg}\max_{x_{i}\in S_{\text{core}}}\text{d}\left(x_{i},C^{k}\right), \end{array}$$

then *C*^*k*+1^=*C*^*k*^∪{*c*_*k*+1_}. Equation (3) shows that *c*_*k*+1_ is the point farthest from the selected center points in the core set *S*_core_. The whole process above is shown in Figure 2.

### 3.2. Phased Assignment

The k-means2o is mainly divided into two stages to complete the clustering. The first stage is to assign cluster label to the core set *S*_core_, and the second stage is to assign cluster label to the noncore set *S*_noncore_. The Tukey's rule is adopted to divide the dataset *X* into sets *S*_core_, *S*_noncore_. Tukey's rule is one of the most robust used techniques for anomaly detection in univariate data [35].

In the first stage, the k-means2o establishes the Tukey's rule for each attribute of the data, and then the judgment results in all dimensions are integrated to determine whether the sample point *x* belongs to the core set *S*_core_.

First, calculate the first quartile *Q*_1_ and third quartile *Q*_3_ on each attribute:

(4) $$\begin{array}{c} Q_{1}^{j}=x_{i}^{j}|i=\text{round}\left(\left(n+1\right)\times 0.25\right),\\ Q_{3}^{j}=x_{i}^{j}|i=\text{round}\left(\left(n+1\right)\times 0.75\right). \end{array}$$

Then, calculate the upper and lower bounds *B*_upper_, *B*_lower_ as follows:

(5) $$\begin{array}{c} B_{\text{lower}}^{j}=Q_{1}^{j}-r\times {\text{IQR}}^{j},\\ B_{\text{upper}}^{j}=Q_{3}^{j}+r\times {\text{IQR}}^{j}, \end{array}$$

where IQR^*j*^=*Q*_3_^*j*^ − *Q*_1_^*j*^ and *r* is a scale factor.

Finally, calculate the core set *S*_core_ and noncore set *S*_noncore_ as follows:

(6) $$\begin{array}{c} S_{\text{core}}=\left\{x_{i}\in X|B_{\text{lower}}^{j}\leq x_{ij}\leq B_{\text{upper}}^{j},\forall j\right\},\\ S_{\text{noncore}}=X-S_{\text{core}}, \end{array}$$

Equation (6) shows that this paper will evaluate each attribute of the data individually, and then integrate all *m* attributes to determine whether it belongs to the core set *S*_core_. As long as any attribute does not satisfy the inequality constraints, it will be judged as belonging to *S*_noncore_. According to equations (3) and (6), it is obvious that *c*_2_ almost will be the point in the noncore set *S*_noncore_, that is, *c*_2_ ∈ *S*_noncore_, and *c*_*i*_, *i* > 2 will also select the point in the noncore set *S*_noncore_ with a high probability.

The scale factor *r* in equation (5) is a predefined adjustable parameter. If you have sufficient prior knowledge of dataset, you can set it depending on experience. If not, it recommends to set *r*=1.5. Although in the field of anomaly detection research, *r*=1.5 is often regarded as the boundary value of the outlier. In cluster analysis, points in *S*_noncore_ cannot be regarded as outliers and discarded, and they still need to be assigned cluster labels. Whether points in *S*_core_ or in *S*_noncore_, in the final clustering result, it is necessary to assign cluster labels which are also one of the goals of cluster analysis. On the 15 real datasets in this paper, each sample has an exact class label, but the *S*_noncore_ of almost all datasets are not empty. After constructing *S*_core_, it is more helpful to obtain a more excellent initial center points. Not only that *S*_core_ effectively assists the selection of the initial center points but also has a positive effect on the update of center points.

When we obtain *S*_core_, use the initial center points selection method described in Section 3.1 to select the initial center points set *C*^*K*^ from *S*_core_, and then use the traditional center points update method of k-means to complete clustering in *S*_core_. Obtain the optimal clustering center points set ${\hat{C}}^{K}$ and clusters ${\bar{C}}_{1},\ldots ,{\bar{C}}_{K}$. The first stage of clustering ends.

(7) $$\begin{array}{c} x_{i}\in {\bar{C}}_{k}\;\Leftrightarrow \text{ d}\left(x_{i},{\hat{C}}^{K}\right)=\text{d}\left(x_{i},{\hat{c}}_{k}\right),x_{i}\in S_{\text{core}}. \end{array}$$

In the second stage, points in *S*_noncore_ will be assigned cluster label. With the help of the optimal clusters ${\bar{C}}_{1},\ldots ,{\bar{C}}_{K}$ obtained in the first stage, determine the cluster label of ∀*x*_*i*_ ∈ *S*_noncore_:

(8) $$\begin{array}{c} x_{i}\in C_{k}\;\Leftrightarrow \text{ d}\left(x_{i},S_{\text{core}}\right)=\text{d}\left(x_{i},{\bar{C}}_{k}\right),x_{i}\in S_{\text{noncore}}, \end{array}$$

where $\text{d}\left(x_{i},S_{\text{core}}\right),\text{d}\left(x_{i},{\bar{C}}_{k}\right)$ are defined in (1), and *C*_*k*_ is the *k* − tk cluster.

**Figure 3:**
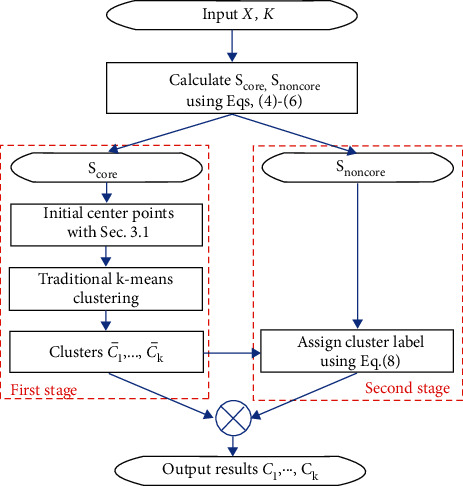
The flow chart of phased assignment.

The whole process above is shown in Figure 3.

### 3.3. Algorithm Flow and Complexity Analysis

The k-means2o algorithm that optimizes the initial center points selection and phased assignment are performed. The algorithm 1 shows its detail process. The steps 1–15 corresponds to the first stage, including that the Step 1 determines *S*_core_, *S*_noncore_, and the Steps 2–4 optimize the initial center points. The Steps 16–19 correspond to the second stage.

According to the detailed steps in algorithm 1, the complexity of k-means2o algorithm is analyzed with data size *n*, attribute *m*, and cluster number *K*. The number of iterations is denoted as *t*, and its maximum value is max_iter. Step 1 generates *S*_core_, *S*_noncore_ with *O*(*nm*). Steps 2–5 select initial center points with *O*(*nK*). Steps 6–13 are a traditional k-means clustering process; however, Step 8 is a new label assignment strategy, so the complexity of these steps becomes *O*(*n*^2^*t*). In summary, the complexity of the k-means2o algorithm is *O*(*n*^2^*t*).

## 4. Performance Analysis of the Proposed Algorithm

In this section, the improved k-means algorithm, k-means2o, testing and verification for clustering performance compared with the well-known k-means ++ [18] which is the most commonly used partition-based algorithm with different initializations of the centroids to reduce the sensitivity. Then, the performance of the k-means2o will be compared with affinity propagation (AP) [2], mean shift (MS) [25], DBSCAN [26]. Although the latter obtain excellent clustering performance on some special datasets, they require to preset one or more important parameter(s), which is a very difficult task. The k-means2o is designed with Python and k-means ++, AP, MS, DBSCAN are called from scikit-learn [36].

### 4.1. Datasets and Evaluation Metrics

A total of 15 real-world datasets used in the experiments were taken from UCI [37]. The data size *n*, attribute *m*, and cluster number *K* are summarized in Table 1 and Table 2 shows 10 synthetic datasets from references [38, 39], where the K1 dataset is synthesized by this paper, see Figure 1. All datasets are publicly available1.

An appropriate and uniform evaluation index is both required and meaningful to compare the different clustering algorithms. Therefore, the quality was measured via the accuracy (ACC), the Adjusted Rand Index (ARI) [40], the Normalized Mutual Information (NMI) [41] and the Fowlkes–Mallows Index (FMI) [42] between the produced clusters and the truth categories. Larger evaluation index values indicate improved clustering performance, and all index upper bounds =1, representing perfectly correct clustering:

(9) $$\begin{array}{c} \text{ACC}=\frac{\sum_{i=1}^{n}\delta \left(\text{label}\_\text{true},\text{map}\left(\text{label}\_\text{pred}\right)\right)}{n},\\ ARI=\frac{RI-E\left[RI\right]}{\max\left(RI\right)-E\left[RI\right]},\\ \text{NMI}=\frac{MI\left(U,V\right)}{\text{mean}\left(H\left(U\right),H\left(V\right)\right)},\\ \text{FMI}=\frac{TP}{\sqrt{\left(TP+FP\right)\left(TP+FN\right)}}. \end{array}$$

where *U*, *V* are predicted label and true label.

### 4.2. Experimental Results and Discussion

The experimental datasets were clustered using k-means ++ and k-means2o. The ACC, ARI, NMI, and FMI of them are listed in Tables 3 and 4, where k-++ represents k-means ++ and k-2o represents k-means2o. The best clustering performance evaluation values are shown in bold, and 1 means that the clustering result is completely correct. The value 0.0000 in the table represents its real metric value <0.0001.

From Table 3, the k-means ++ and k-means2o simultaneously obtained the maximum FMI value for 8 of the 15 datasets. This shows that the two algorithms have the same performance, and further performance comparison and analysis of other evaluation indicators are required. From the view of ARI in Table 3, the most significant and direct conclusion is that the k-means2o outperforms the k-means ++ on most datasets, and the performance of the two algorithms is also very close on a few datasets that are inferior to k-means ++. Specifically, the k-means2o achieved the maximum ARI value for 10 of the 15 datasets, as well as the NMI and it obtained the same result, and the k-means ++ achieved the best clustering performance only on 6 datasets in ARI, as well as in NMI. For banknote, iris, wine datasets, the k-means2o is only inferior to k-means ++ with a small gap. For ACC evaluation, it comes to the exact same conclusion as NMI and ARI, that is, the k-means2o clustering performance is better than the k-means ++.

**Figure 4:**
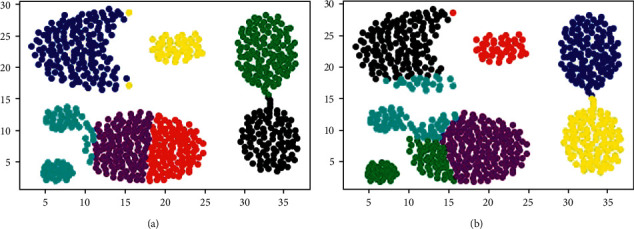
Clustering results on aggregation dataset: (a) k-means ++; (b) k-means2o.

For the synthetic datasets in Table 2, the four evaluation metrics in Table 4 show that k-means ++ and k-means2o have similar clustering performance. For datasets with spherical cluster distribution, such as D31, R15, S1, and S3, the clustering results of the two algorithms are close to the real cluster partition, while for datasets with nonspherical distribution such as spiral, flame, circlesA3, the clustering performance of them drops sharply. When the size of the distribution area of spherical clusters is significantly different, the performance difference between k-means ++ and k-means2o can be revealed. For example, in the aggregation dataset, the two algorithms' clustering results are shown in Figure 4. The evaluation values of ARI, NMI, and FMI all show that k-means ++ is better than k-means2o, but ACC gives the opposite conclusion.  Figure 4(a) shows that k-means ++ selects seven center points in six real clusters, and two different clusters (green points in the figure) are wrongly classified into the same cluster.  Figure 4(b) shows that k-means2o can select center points in seven real clusters, respectively.

Further, the performance of the k-means2o will be compared with AP, MS, and DBSCAN. The ARI and NMI of these algorithms are listed in Table 5, and the ACC and FMI are listed in Table 6. The values larger than the one of the k-means2o are marked in bold. The three comparison algorithms all use default parameters. Considering better performance, the data are normalized here. From the perspective of ARI values, compared with AP, MS, and DBSACN, the k-means2o obtained better clustering performance on 12,14,13 datasets, respectively. The evaluation results of NMI are similar to ARI, except for the AP algorithm. The AP's measurement results of NMI and ARI are very different, which may be tied to the number of error clusters given by the AP algorithm. The ACC evaluation conclusion is consistent with ARI, but FMI and NMI reach opposite conclusions. For the MS algorithm, its FMI value is better than k-means2o algorithm in 9 out of 15 datasets, while for the AP algorithm, its FMI value on all datasets is smaller than k-means2o algorithm. Based on the four evaluation metrics, the k-means2o algorithm is superior to the comparison methods in at least three of these metrics on most datasets. Therefore, k-means2o has better clustering performance.

As for the abnormal conclusion given by a certain evaluation metric for a specific algorithm, for example, the NMI evaluation metric for the AP algorithm, the FMI evaluation metric for the MS algorithm, it may be caused by too many or too few clusters.  Table 7 shows that the AP and MS algorithms give the wrong number of clusters on any datasets, and the former far exceeds the true number of clusters, while the latter divides more than half of the datasets into one cluster. Undeniably, the AP, MS, and DBSCAN algorithms provide a method to identify the number of clusters. If the parameters for the AP algorithm, damping factor, and preference value are carefully adjusted, it maybe achieves better clustering performance in these real-world datasets. In those clustering algorithms that contain parameters, careful selection of parameters is often time-consuming and requires prior knowledge. Therefore, these algorithms have poor universality.

**Figure 5:**
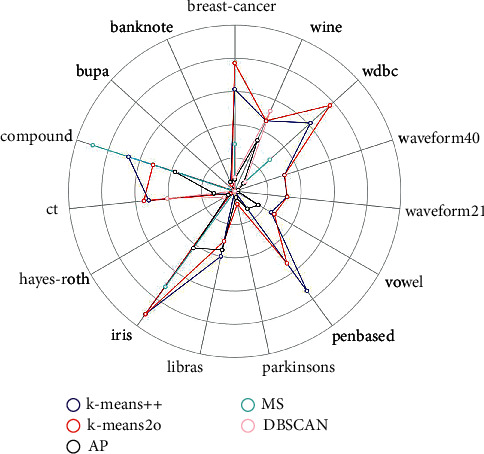
Radar chart of ARI values on the real-world datasets.

The performance of all five algorithms can be directly compared in Figure 5. In this radar chart, each axis represents a dataset, and its value is the cluster evaluation ARI value. According to the previous analysis, the k-means2o has the best performance, and its corresponding red line in the radar chart reaches the maximum value on more polar axes, that is, farther away from the center point.

### 4.3. Comparative Analysis of Different Initialization Methods

In this subsection, the effects of three different initialization methods on the performance of the k-means clustering algorithm are compared. These three methods are represented by Random, *D*^2^-sampling, New respectively, see the header of Table 8. Random means randomly initializing the center point. *D*^2^-sampling means assigning a selection probability to each noncenter point and randomly selecting the center point. New means the center point initialization optimization method proposed in this paper. In fact, the k-means algorithm based on *D*^2^-sampling is the famous k-means ++ algorithm.

The initial center points optimization plays an important role in the performance improvement of k-means2o. However, Table 8 shows that only using the initialization method proposed in this paper cannot improve the clustering performance. From the evaluation value of ARI, the optimal initialization method is *D*^2^-sampling, followed by Random, and the worst is New which is the initialization method proposed in this paper. Except for tiny numerical differences on individual datasets, the NMI evaluation shows similar conclusions. Combined with the conclusion of k-means2o performance improvement, it is the combination of initial center point optimization and phased assignment that improves the performance of k-means2o, not just the center points optimization.

### 4.4. Impact Analysis of Core and Noncore Sets

This paper uses Tukey's rule to realize the division of *S*_core_ and *S*_noncore_. Therefore, a scale factor *r* needs to be given. Tukey's rule comes from the field of anomaly detection. Generally, the scale factor is set to 1.5. Points that do not meet the conditions of the scale factor are called outliers. In most cases, these points are directly abandoned. This idea is introduced into cluster analysis and used in the data preprocessing stage. As a result, the points detected as abnormal will be discarded and not assign cluster label. There will be great hidden trouble in this way.  Table 9 shows the number of elements in *S*_core_ and *S*_noncore_ in 15 real-world datasets when *r*=1.5. Except that the *S*_noncore_ of compound dataset is empty, the *S*_noncore_ of the remaining 14 datasets are not empty. However, as well as we known, all points in these datasets are labeled with class labels. Therefore, it is unreasonable to abandon these suspected outliers simply and rudely. For this reason, this paper proposes a two-stage assignment method, whose first stage assigns cluster label to the points in *S*_core_ and second stage assigns the points in the *S*_noncore_. For the compound dataset, the empty *S*_noncore_ indicates that Tukey's rule has no effect on this dataset and will directly lead to the failure of the second stage assignment.

**Figure 6:**
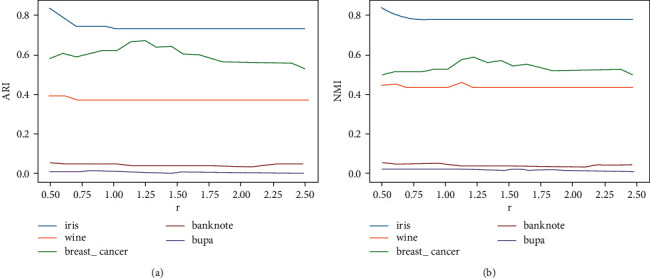
Clustering evaluation for different *r* on five datasets: (a) ARI curve and (b) NMI curve.

The k-means2o algorithm relies on a predefined scale factor *r*, so it is necessary to perform a sensitivity test of this parameter. Therefore, we took the iris, wine, breast_cancer, banknote, and bupa datasets as an example to investigate the effects of different *r* on ARI and NMI, as shown in Figure 6. Its shows that the ARI and NMI curves of the five datasets do not fluctuate drastically, so the clustering performance of the k-means2o algorithm based on the scale factor *r* is relatively robust. Nevertheless, the scale factor *r* still has a slight impact on the clustering performance. For example, in the iris dataset, when *r*=0.5, its ARI and NMI values reach 0.8340 and 0.8191, respectively. This clustering result is better than k-means ++, see Table 3 (the values are ARI =0.7302 and NMI =0.7581).

In the above analysis, the k-means2o outperforms k-means ++, AP, MS, and DBSCAN. Combined with the fact, almost all *S*_*noncore*_ of these datasets in Table 9 are nonempty. These results show that the combination optimization of the initial center point and the core subset works and improves the k-means clustering performance.

## 5. The Application of K-Means2o

In this section, the k-means2o is applied into cluster to analyze the airline seat selection dataset provided by Neusoft. According to the meaning of clustering, the samples in the same cluster are as similar as possible, and the samples between different clusters are as dissimilar as possible. If most samples in the same cluster have a certain property, it can be inferred that other samples in the same cluster are also most likely to have the same property. If the most passengers in the cluster are willing to accept some of the personalized recommendation service, such as paying for seat selection, the same service should be recommended to other passengers in the cluster, and a clearer audience group will increase the personalized recommendation service success rate. For the airline seat selection dataset, the appropriate clusters number is required to be determined first.

**Figure 7:**
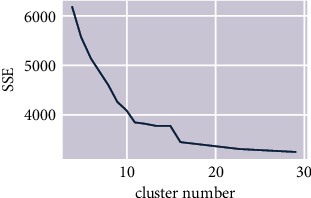
Silhouette coefficient of the k-means2o algorithm on passenger seat selection dataset.

The silhouette coefficient is a simple and effective method to determine the appropriate clusters number for the k-means algorithm. The silhouette coefficient of the k-means2o algorithm on this dataset is shown in Figure 7. The figure shows that the SSE change tends to be gentle from 16 clusters. Therefore, the optimal number of clusters would be selected as 16. Then, the k-means2o is applied and divides the data into 16 clusters. The number of passengers in each cluster is shown in the column named as size in Table 10. The 3rd, 4th, and 5th columns of Table 10 (payment, no-payment, payment ratio), respectively, show the number of paid passengers, the number of nonpaid passengers and the proportion of paid ones in the airline seat selection. The absolute deviation rate adr in the last column is defined as follows:

(10) $$\begin{array}{c} {\text{adr}}_{c}=\frac{\left|r_{c}-r\right|}{r}, \end{array}$$

where *r*_*c*_ is the payment rate in cluster *c* and *r* is the payment rate in the dataset. The larger the adr value, the more significant the difference between the payment behavior of passengers in the cluster and the whole dataset.

The clustering results show that the number of passengers in each cluster is not close. The cluster with the largest number of passengers is *C*0, with 2580, while the smallest one is *C*13, with 379.

**Figure 8:**
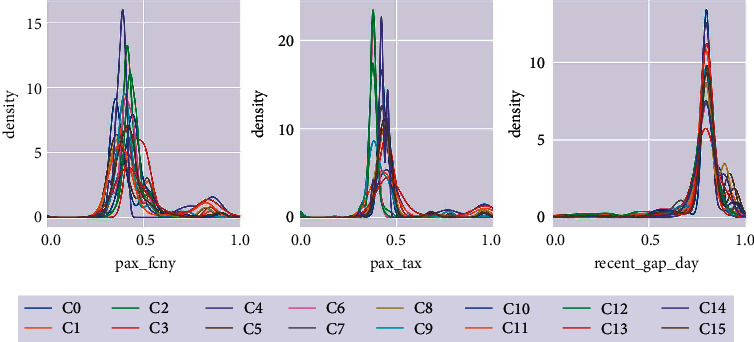
Kernel density estimation curves of pax_fcny, pax_tax, and recent_gap_day attributes.

Further, the significant differences are explored between clusters.  Figure 8 shows the kernel density estimation curves of three attributes, pax_fcny, pax_tax, recent_gap_day. On the whole, these curves in each cluster are not completely coincident, and there are significant differences, which show that the data distribution of each cluster is different. This conclusion is consistent with the expectation of cluster analysis, that is, the samples between clusters are dissimilar as much as possible. From a single attribute point of view, the discrimination of pax_fcny attribute is the most significant, with different mean point, peak point, and data span. Followed by pax_tax attribute. The third one is recent_gap_day attribute. Its mean and span are very similar, but the peak point is still different. The difference of peak points indicates that there are differences in the concentration of data distribution in the cluster. The larger the peak value, more points are distributed near the mean value.

Table 10 discusses the k-means2o algorithm clustering results of the airline seat selection dataset from the similarities within clusters and dissimilarities between the clusters. The clustering results will be a good reference basis for customer grouping. Air passenger grouping will enable the decision-makers to more accurately find the audience of the personalized recommendation service, such as payment for airline seat selection. The dataset shows the label of payment for airline seat selection. The adr value of each cluster is greater than 12%, which is significantly different from the payout rate of the entire dataset of 6.29%. The cluster with the largest adr value is C13, reaching 66.45%, and the one with the smallest *adr* value is c5, reaching 12.56%. These results show that passenger payment behavior within clusters is more agglomerated compared to the entire dataset. Since the payment rate of C13 is 2.11%, it is a reverse difference. In other words, the *adr* = 66.45% indicates that passengers in C13 are extremely unwilling to pay for seat selection, and the willingness to pay is significantly lower than the overall level. In 9 of the 16 clusters, the ratio of paying for airline seat selection exceeds 5%. According to the precise recommendation or personalized marketing strategy, enterprises should pay more attention to the passengers in these nine clusters, and their marketing is more likely to succeed. Compared with the passengers in other clusters, the ones in these clusters will be more willing to accept such recommendations and enhance their stickiness. On designing a recommendation system, this clustering result will become a good auxiliary prior information.

## 6. Conclusion

In this paper, two optimization methods for k-means are initial center points selection and phased assignment were proposed, and then the improved k-means algorithm, k-means2o, were proposed. In contrast to the previously introduced algorithms, k-means ++, K-MC^2^, and AFK-MC^2^, the new initial center points selection optimization redefines the first center point selection strategy and the subsequent center point incremental strategy. The phased assignment optimization adopted the Tukey's rule to divide dataset into core and noncore sets, then two assignment strategies were proposed corresponding to the core and noncore sets, respectively. These two optimization methods complement each other to form combinatorial optimization. The experimental results on 15 real-world and 10 synthetic datasets show that the k-means2o outperforms its main competitor k-means ++, and under the same setting conditions, namely using the default parameters, the clustering performance of k-means2o is better than affinity propagation, mean shift, and DBSCAN.

The improved k-means algorithm, k-means2o, is applied to analyze the airline seat selection dataset. Combined with the data label of paying for seat selection, the clustering results realize customer grouping, and find suitable audience group for the recommendation of seat selection services. Through the analysis of the newly defined absolute deviation rate *adr* index, the appropriate groups for service recommendation are found, and the groups that are not suitable for recommendation are distinguished. Therefore, the airline enterprises can use limited resources to promote the groups with high-payment willingness, improve the success rate, and avoid promoting seat selection services to the groups with low-payment willingness which not only wastes resources but also causes passengers' disgust.

After a lot of experimental tests, the k-means2o algorithm, like other algorithms, cannot be adapted to all fields and situations, such as high-dimensional sparse data. If the data are a huge number of attributes or higher dimensions, it will easily lead to fewer samples in *S*_*core*_, and in extreme cases, it may be less than the number of clusters. The Olivetti Face image data with 112*∗*92=10304 dimension have been tested and found that *|S*_*core*_*|* < 40, that is, the number of samples in the core set is less than the number of clusters; therefore, the clustering fails. Due to the division of the core and noncore sets, the k-means2o algorithm is not suitable for huge number of attributes or higher dimensions. We will continue to study this problem and hope to solve this problem in the future.

## Figures and Tables

**Algorithm 1:**
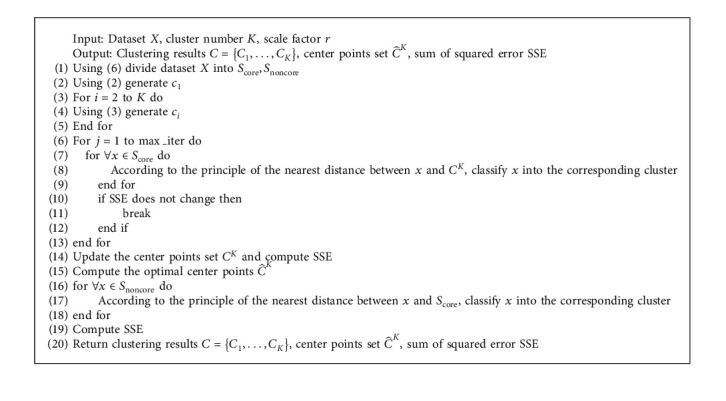
k-means2o.

**Table 1:** Real-world datasets.

Dataset	*n*	m	*K*	Dataset	*n*	m	*K*
Breast-cancer	569	30	2	Banknote	1372	4	2
Bupa	345	6	2	Compound	399	2	6
Ct	221	36	2	Hayes-roth	132	5	3
Iris	150	4	3	Libras	360	90	15
Parkinsons	195	22	2	Penbased	10992	16	10
Vowel	990	10	11	Waveform21	5000	21	3
Waveform40	5000	40	3	Wdbc	569	30	2
Wine	178	13	3				

**Table 2:** Synthetic datasets.

Dataset	*n*	m	*K*	Dataset	*n*	m	*K*
Aggregation	788	2	7	circlesA3	300	2	3
D31	3100	2	31	Flame	240	2	2
Jain	373	2	2	K1	262	2	5
R15	600	2	15	S1	5000	2	15
S3	5000	2	15	Spiral	312	2	3

**Table 3:** Clustering results of k-means ++ and k-means2o on real-world datasets.

Dataset	ACC		ARI		NMI		FMI	
	k-++	k-2o	k-++	k-2o	k-++	k-2o	k-++	k-2o
Breast-cancer	0.8541	**0.8910**	0.4914	**0.6062**	0.4647	**0.5276**	0.7915	**0.8286**
Banknote	**0.6122**	0.5954	**0.0485**	0.0356	**0.0303**	0.0239	**0.5517**	0.5231
Bupa	**0.8550**	0.5768	0.0000	**0.0058**	0.0000	**0.0112**	**0.6192**	0.5136
Compound	**0.6566**	0.6365	**0.5378**	0.5043	**0.7191**	0.6557	**0.6422**	0.6181
Ct	0.8235	**0.8325**	0.4160	**0.4399**	0.3296	**0.3485**	0.7078	**0.7199**
Hayes–Roth	0.4393	**0.4469**	0.0202	**0.0226**	0.0287	**0.0317**	0.3501	**0.3519**
Iris	**0.8933**	**0.8933**	**0.7302**	**0.7302**	**0.7581**	**0.7581**	**0.8208**	**0.8208**
Libras	0.4277	**0.4416**	**0.3199**	0.2760	**0.6066**	0.5716	**0.3734**	0.3389
Parkinsons	**0.7230**	0.6307	0.0000	**0.0625**	0.0000	**0.0493**	**0.7444**	0.5889
Penbased	**0.7674**	0.6035	**0.5992**	0.4907	**0.6927**	0.6723	**0.6412**	0.5582
Vowel	0.3636	**0.3645**	0.2028	**0.2204**	0.4141	**0.4337**	0.2789	**0.2868**
Waveform21	0.5016	**0.5018**	0.2536	**0.2547**	0.3622	**0.3654**	0.5039	**0.5047**
Waveform40	0.5146	**0.5160**	0.2516	**0.2530**	0.5023	**0.5035**	0.3605	**0.3632**
Wdbc	0.8541	**0.8910**	0.4914	**0.6062**	0.4647	**0.5276**	0.7915	**0.8286**
Wine	**0.7022**	**0.7022**	**0.3711**	0.3675	**0.4287**	0.4164	**0.5835**	0.5809
Maximum	7	10	6	10	6	10	8	8

The best clustering performance evaluation values are shown in bold.

**Table 4:** Clustering results of k-means ++ and k-means2o on synthetic datasets.

Dataset	ACC		ARI		NMI		FMI	
	k-++	k-2o	k-++	k-2o	k-++	k-2o	k-++	k-2o
Aggregation	0.7855	**0.8680**	**0.7624**	0.7438	**0.8792**	0.8373	**0.8159**	0.7992
CirclesA3	0.5833	**0.5866**	0.1283	**0.1311**	0.3616	**0.3633**	0.4903	**0.4915**
D31	**0.9764**	0.9290	**0.9522**	0.9059	**0.9669**	0.9498	**0.9538**	0.9090
Flame	**0.8375**	**0.8375**	**0.4534**	**0.4534**	**0.3987**	**0.3987**	**0.7363**	**0.7363**
Jain	**0.7855**	**0.7855**	**0.3241**	**0.3241**	**0.3690**	**0.3690**	**0.7005**	**0.7005**
K1	0.7824	**0.9923**	0.7318	**0.9809**	0.8160	**0.9765**	0.7962	**0.9847**
R15	**0.9966**	**0.9966**	**0.9927**	**0.9927**	**0.9942**	**0.9942**	**0.9932**	**0.9932**
S1	**0.9938**	0.9936	**0.9867**	0.9863	**0.9866**	0.9861	**0.9876**	0.9872
S3	**0.8568**	**0.8568**	**0.7270**	**0.7270**	0.7959	**0.7962**	**0.7453**	**0.7453**
Spiral	**0.3461**	**0.3461**	**0.0000**	**0.0000**	**0.0007**	0.0005	0.3276	**0.3277**
Maximum	7	8	8	7	7	6	7	7

The best clustering performance evaluation values are shown in bold.

**Table 5:** ARI and NMI evaluation results on real-world datasets with AP, MS, DBSCAN.

Dataset	ARI				NMI			
	AP	MS	DBSCAN	k-2o	AP	MS	DBSCAN	k-2o
Breast-cancer	0.0574	0.2275	0.0687	0.6062	0.2692	0.2439	0.0415	0.5276
Banknote	0.0491	0.0000	0.0000	0.0356	0.2973	0.0000	0.0000	0.0239
Bupa	0.0000	0.0000	0.0037	0.0058	0.0287	0.0237	0.0130	0.0112
Compound	0.3023	0.7189	0.0000	0.4133	0.6289	0.7692	0.0000	0.6240
Ct	0.1006	0.0000	0.3271	0.4399	0.2529	0.0601	0.2563	0.3485
Hayes-Roth	0.0350	0.0000	0.0589	0.0226	0.2010	0.0000	0.1168	0.0317
Iris	0.3381	0.5681	0.0000	0.7302	0.5706	0.7336	0.0000	0.7581
Libras	0.2882	0.0000	0.0058	0.2760	0.6375	0.0000	0.1449	0.5716
Parkinsons	0.0305	0.0000	0.0000	0.0625	0.1889	0.0641	0.0227	0.0493
Penbased	0.1037	0.0000	0.0008	0.4907	0.5929	0.0000	0.0380	0.6723
Vowel	0.1315	0.0000	0.0000	0.2204	0.5525	0.0000	0.0000	0.4337
Waveform21	0.0168	0.0000	0.0000	0.2547	0.2345	0.0000	0.0003	0.3654
Waveform40	0.0187	0.0000	0.0000	0.2530	0.2052	0.0000	0.0000	0.3632
Wdbc	0.0574	0.2275	0.0687	0.6062	0.2692	0.2439	0.0415	0.5276
Wine	0.2689	0.0000	0.4228	0.3675	0.5264	0.0000	0.5263	0.4164
Number	3	1	2	—	8	2	1	—

**Table 6:** ACC and FMI evaluation results on real-world datasets with AP, MS, DBSCAN.

Dataset	ACC				FMI			
	AP	MS	DBSCAN	k-2o	AP	MS	DBSCAN	k-2o
Breast-cancer	0.1353	0.7153	0.6626	0.8910	0.2491	0.7117	0.6775	0.8286
Banknote	0.1013	0.5554	0.5554	0.5954	0.2230	0.7112	0.7112	0.5231
Bupa	0.0841	0.5188	0.9913	0.5768	0.1540	0.6224	0.7073	0.5136
Compound	0.3584	0.7393	0.3960	0.6365	0.4530	0.8159	0.4972	0.6181
Ct	0.2172	0.4977	0.7873	0.8325	0.3267	0.6724	0.6633	0.7199
Hayes-Roth	0.1515	0.3864	0.3561	0.4469	0.1981	0.5876	0.3436	0.3519
Iris	0.4133	0.6667	0.3333	0.8933	0.5145	0.7715	0.5735	0.8208
Libras	0.3889	0.0667	0.1278	0.4416	0.3333	0.2531	0.2429	0.3389
Parkinsons	0.1590	0.6513	0.6154	0.6307	0.2465	0.6857	0.6436	0.5889
Penbased	0.1018	0.1041	0.1195	0.6035	0.2416	0.3164	0.3080	0.5582
Vowel	0.1657	0.0909	0.0909	0.3645	0.2266	0.3000	0.3000	0.2868
Waveform21	0.0368	0.3392	0.3374	0.5018	0.1067	0.5773	0.5613	0.5047
Waveform40	0.0650	0.3384	0.3384	0.5160	0.1124	0.5773	0.5773	0.5035
Wdbc	0.1353	0.7153	0.6626	0.8910	0.2491	0.7117	0.6775	0.8286
Wine	0.3427	0.3989	0.6966	0.7022	0.4604	0.5813	0.6482	0.5809
Number	0	2	1	—	0	9	7	—

**Table 7:** The number of real and 3 algorithms predicted clusters in the real-world datasets.

Dataset	AP	MS	DBSCAN	Real
Breast-cancer	43	12	2	2
Bupa	32	14	2	2
Ct	20	7	2	2
Iris	9	2	1	3
Parkinsons	21	5	2	2
Vowel	85	1	1	11
Waveform40	157	1	1	3
Wine	14	1	3	3
Banknote	45	1	1	2
Compound	15	3	1	6
Hayes-roth	16	1	4	3
Libras	30	1	6	15
Penbased	199	1	7	10
Waveform21	148	1	2	3
Wdbc	43	12	2	2

**Table 8:** Results of different initialization methods of k-means algorithm.

Dataset	ARI			NMI		
	Random	*D* ^2^-Sampling	New	Random	*D* ^2^-Sampling	New
Breast-cancer	0.4914	0.4914	0.4914	0.4914	0.4914	0.4914
Banknote	0.0485	0.0485	0.0485	0.0485	0.0485	0.0485
Bupa	0.0000	0.0000	0.0000	0.0000	0.0001	0.0001
Compound	0.5328	0.5378	0.4133	0.7220	0.7191	0.6240
Ct	0.4160	0.4160	0.0000	0.3296	0.3296	0.0219
Hayes-Roth	0.0160	0.0202	0.0202	0.0250	0.0287	0.0287
Iris	0.7302	0.7302	0.7302	0.7581	0.7581	0.7581
Libras	0.3062	0.3199	0.2868	0.5896	0.6066	0.5767
Parkinsons	0.0853	0.0000	0.0001	0.0505	0.0001	0.0001
Penbased	0.5442	0.5992	0.4265	0.6835	0.6927	0.6486
Vowel	0.2180	0.2028	0.2058	0.4332	0.4141	0.4067
Waveform21	0.2536	0.2536	0.2535	0.3622	0.3622	0.3622
Waveform40	0.2516	0.2516	0.2515	0.3605	0.3605	0.3605
Wdbc	0.4914	0.4914	0.4914	0.4647	0.4647	0.4647
Wine	0.3711	0.3711	0.3711	0.4287	0.4287	0.4287
Maximum	11	13	7	11	12	9

**Table 9:** The number of core and noncore subsets elements |*S*_core_|, |*S*_*noncore*_| in k-means2o clustering.

Dataset	|*S*_core_|	|*S*_*noncore*_|	Dataset	|*S*_core_|	|*S*_*noncore*_|
Breast-cancer	398	171	Banknote	1280	92
Bupa	280	65	Compound	399	0
Ct	164	57	Hayes-roth	102	30
Iris	146	4	Libras	356	4
Parkinsons	148	47	Penbased	10482	510
Vowel	960	30	waveform21	4740	260
Waveform40	4116	884	Wdbc	398	171
Wine	161	17			

**Table 10:** The airline seat selection dataset clustering result.

Cluster	Size	Payment	No-payment	Payment ratio (%)	adr (%)
*C*0	2580	185	2395	7.17	13.99
*C*1	1771	127	1644	7.17	13.99
*C*2	1139	30	1109	2.63	58.19
*C*3	1256	107	1149	8.52	35.45
*C*4	1596	43	1553	2.69	57.23
*C*5	1582	112	1470	7.08	12.56
*C*6	761	22	739	2.89	54.05
*C*7	1935	166	1769	8.58	36.41
*C*8	1930	140	1790	7.23	14.94
*C*9	1623	67	1556	4.13	34.34
*C*10	2023	176	1847	8.70	38.31
*C*11	695	21	674	3.02	51.99
*C*12	1438	58	1380	4.03	35.93
*C*13	379	8	371	2.11	66.45
*C*14	909	67	842	7.37	17.17
*C*15	1815	146	1669	8.04	27.82

## Data Availability

The data are available at https://gitee.com/ydh-usx/k-means2o-data/tree/master/data.

## References

[1] Han J., Pei J., Kamber M. (2011). *Data Mining: Concepts and Techniques*.

[2] Frey B. J., Dueck D. (2007). Clustering by passing messages between data points. *Science*.

[3] Rodriguez A., Laio A. (2014). Clustering by fast search and find of density peaks. *Science*.

[4] Parmar M., Wang D., Tan A. H., Miao C., Jiang J., Zhou Y. A novel density peak clustering algorithm based on squared residual error.

[5] Parmar M., Wang D., Zhang X. (2019). REDPC: a residual error-based density peak clustering algorithm. *Neurocomputing*.

[6] Parmar M. D., Pang W., Hao D. (2019). FREDPC: a feasible residual error-based density peak clustering algorithm with the fragment merging strategy. *IEEE Access*.

[7] Xie H., Zhang L., Lim C. P. (2019). Improving k-means clustering with enhanced firefly algorithms. *Applied Soft Computing*.

[8] Shah N., Patel D., Fränti P. (2021). k-means image segmentation using mumford–shah model. *Journal of Electronic Imaging*.

[9] Khan A. R., Khan S., Harouni M., Abbasi R., Iqbal S., Mehmood Z. (2021). Brain tumor segmentation using k-means clustering and deep learning with synthetic data augmentation for classification. *Microscopy Research and Technique*.

[10] Moubayed A., Injadat M., Shami A., Lutfiyya H. (2020). Student engagement level in an e-learning environment: clustering using k-means. *American Journal of Distance Education*.

[11] Qian X., Di Renzo M., Eckford A. (2021). K-means clustering-aided non-coherent detection for molecular communications. *IEEE Transactions on Communications*.

[12] Xu Z., Shen D., Nie T., Kou Y., Yin N., Han X. (2021). A cluster-based oversampling algorithm combining smote and k-means for imbalanced medical data. *Information Sciences*.

[13] Liu H., Wu J., Liu T., Tao D., Fu Y. (2017). Spectral ensemble clustering via weighted k-means: theoretical and practical evidence. *IEEE Transactions on Knowledge and Data Engineering*.

[14] Aldino A. A., Darwis D., Prastowo A. T., Sujana C. (2021). Implementation of k-means algorithm for clustering corn planting feasibility area in south lampung regency. *In Journal of Physics: Conference Series*.

[15] Chen Z.-S., Zhang X., Pedrycz W., Wang X.-J., Chin K.-S., Martínez L. (2021). K-means clustering for the aggregation of HFLTS possibility distributions: N-two-stage algorithmic paradigm. *Knowledge-Based Systems*.

[16] Fränti P., Sieranoja S. (2019). How much can k-means be improved by using better initialization and repeats?. *Pattern Recognition*.

[17] Huang S., Kang Z., Xu Z., Liu Q. (2021). Robust deep k-means: an effective and simple method for data clustering. *Pattern Recognition*.

[18] Vassilvitskii S. K-means: The advantages of careful seeding.

[19] Warnock-Smith D., O’Connell J. F., Maleki M. (2017). An analysis of ongoing trends in airline ancillary revenues. *Journal of Air Transport Management*.

[20] Chiambaretto P. (2021). Air passengers’ willingness to pay for ancillary services on long-haul flights. *Transportation Research Part E: Logistics and Transportation Review*.

[21] Vinod B. (2021). Airline revenue planning and the covid-19 pandemic. *Journal of Tourism Futures*.

[22] Gunardi G., Moody R. S., Martono H. k Covid-19: the impact on air transportation tariff in Indonesia.

[23] Bachem O., Lucic M., Hamed Hassani S., Krause A. Approximate k-means++ in sublinear time.

[24] Bachem O., Lucic M., Hassani H., Krause A. (2016). Fast and provably good seedings for k-means. *Advances in Neural Information Processing Systems*.

[25] Comaniciu D., Meer P. (2002). Mean shift: a robust approach toward feature space analysis. *IEEE Transactions on Pattern Analysis and Machine Intelligence*.

[26] Schubert E., Sander J., Ester M., Kriegel H. P., Xu X. (2017). Dbscan revisited, revisited: why and how you should (still) use dbscan. *ACM Transactions on Database Systems*.

[27] Celebi M. E., Kingravi H. A., Vela P. A. (2013). A comparative study of efficient initialization methods for the k-means clustering algorithm. *Expert Systems with Applications*.

[28] Zhou Y., Hong Y., Cai X. A novel k-means algorithm for clustering and outlier detection.

[29] Sungjin I., Montazer Qaem M., Moseley B., Sun X., Zhou R. Fast noise removal for k-means clustering.

[30] Gan G., Ng M. K. P. (2017). K-means clustering with outlier removal. *Pattern Recognition Letters*.

[31] Peter O., Twala B. K-means-sharp: modified centroid update for outlier-robust k-means clustering.

[32] Shrifan N. H. M. M., Akbar M. F., Mat Isa N. A. (2021). An Adaptive Outlier Removal Aided K-Means Clustering Algorithm Journal of King Saud University-Computer and Information Sciences.

[33] Yu D., Liu G., Guo M., Liu X., Yao S. (2019). Density peaks clustering based on weighted local density sequence and nearest neighbor assignment. *IEEE Access*.

[34] Wang L., Sun W., Han X. (2021). An improved integrated clustering learning strategy based on three-stage affinity propagation algorithm with density peak optimization theory. *Complexity*.

[35] Huyghues-Beaufond N., Tindemans S., Falugi P., Sun M., Strbac G. (2020). Robust and automatic data cleansing method for short-term load forecasting of distribution feeders. *Applied Energy*.

[36] Pedregosa F., Varoquaux G., Gramfort A. (2011). Scikit-learn: machine learning in Python. *Journal of Machine Learning Research*.

[37] Dua D., Graff C. (2017). *UCI Machine Learning Repository*.

[38] Liu L., Yu D. (2020). Density peaks clustering algorithm based on weighted k-nearest neighbors and geodesic distance. *IEEE Access*.

[39] Yu D., Liu G., Guo M., Liu X. (2018). An improved k-medoids algorithm based on step increasing and optimizing medoids. *Expert Systems with Applications*.

[40] Steinley D. (2004). Properties of the hubert-arable adjusted rand index. *Psychological Methods*.

[41] Xuan Vinh N., Epps J., Bailey J. (2010). Information theoretic measures for clusterings comparison: variants, properties, normalization and correction for chance. *Journal of Machine Learning Research*.

[42] Fowlkes E. B., Mallows C. L. (1983). A method for comparing two hierarchical clusterings. *Journal of the American Statistical Association*.

